# Effects of Implementing an Enhanced Recovery After Cardiac Surgery Protocol with On-Table Extubation on Patient Outcome and Satisfaction—A Before–After Study

**DOI:** 10.3390/jcm14020352

**Published:** 2025-01-08

**Authors:** Adelina Werner, Hannah Conrads, Johanna Rosenberger, Marcus Creutzenberg, Bernhard Graf, Maik Foltan, Sebastian Blecha, Andrea Stadlbauer, Bernhard Floerchinger, Maria Tafelmeier, Michael Arzt, Christof Schmid, Diane Bitzinger

**Affiliations:** 1 Department of Anesthesiology, University Hospital of Regensburg, Franz-Josef-Strauss-Allee 11, 93053 Regensburg, Germany; 2 Department of Cardiothoracic Surgery, University Hospital Regensburg, Franz-Josef-Strauss-Allee 11, 93053 Regensburg, Germany; 3 Department of Internal Medicine II, University Hospital Regensburg, Franz-Josef-Strauss-Allee 11, 93053 Regensburg, Germany

**Keywords:** enhanced recovery after surgery (ERAS), on-table extubation, elective cardiac surgery, cardiac anesthesia, nausea and vomiting, postoperative cognitive dysfunction, patient satisfaction

## Abstract

**Background/Objectives**: Enhanced recovery after surgery (ERAS) protocols aim to improve clinical outcomes, shorten hospital length of stay (LOS), and reduce costs through a multidisciplinary perioperative approach. Although introduced in colorectal surgery, they are less established in cardiac surgery, especially in combination with on-table extubation (OTE). This study evaluates the impact of a novel ERAS concept with OTE (RERACS) in elective aortic-valve-replacement and coronary bypass surgery. **Methods**: In a monocentric study, we compared a prospective RERACS-group (*n* = 114) to a retrospective control group (*n* = 119) (TRIAL Registration (DRKS00031402). The RERACS concept contained multiple perioperative treatment measures such as respiratory training, short fasting, and OTE. The control group received standard care. **Results**: Primary endpoint: postoperative LOS. Secondary measurements: length of postoperative vasoactive drug support, duration of mechanical ventilation, complication rate, and patient satisfaction on the second postoperative day. RERACS patients showed significantly shorter postoperative length of stay (ICU: 40 ± 34 h vs. 56 ± 51 h, *p* = 0.005; hospital: 9 ± 4 d vs. 11 ± 6 d, *p* = 0.028), lower nosocomial infection rates (24% vs. 40%), fewer cases of postoperative cognitive dysfunction ((subsyndromal) delirium 40% vs. 57%), reduced nausea and vomiting (14.9% vs. 32.8%), and faster weaning from catecholamines (22 ± 30 h vs. 42 ± 48 h, *p* < 0.001), as well as high patient satisfaction. **Conclusions**: Our study indicated that an ERAS concept with OTE is safe and associated with faster and improved recovery, including lower catecholamine requirements, reduced LOS, and high patient satisfaction in low-risk cardiac surgery.

## 1. Introduction

High-quality, resource-aware and individualized perioperative care is outcome-relevant in the modern era of cardiac surgery [1]. Despite numerous surgical and technological advancements throughout the decades, postoperative complication rates remain of high concern in major surgical interventions [2]. The Enhanced Recovery After Surgery (ERAS)-Society aims to enhance and accelerate perioperative patient care. Originally focused solely on colorectal surgery, the ERAS-Society now publishes and revises ERAS-guidelines for many different fields of surgery [3,4].

In 2019 the first Enhanced Recovery After Cardiac Surgery (ERACS)-guidelines were published, most importantly by the ERAS-Society [5], the French Society of Anesthesia and Intensive Care [6], and an interdisciplinary expert consensus statement [7]. Although these recommendations have been studied individually, there is still a lack of research on the implementation of an entire ERAS-treatment-bundle in cardiac surgery. This might be due to the fragile patient population with many older and highly comorbid individuals.

Recently, there has been a growing debate about the potential benefits of on-table extubation (OTE) in cardiac surgical patients, particularly its role in enhancing clinical outcomes [8,9,10] compared to the current recommendation of extubation within 6 h after surgery. In the past decades, the use of high-dose opioids in patients undergoing cardiac surgery imposed the need for postoperative overnight mechanical ventilation and led to a relatively high incidence of respiratory complications as well as delays in neurocognitive recovery [11]. Delayed extubation is performed for a multitude of reasons, such as concerns about early postoperative bleeding and caution of combined respiratory and circulatory failure. It is still not properly defined which factors contribute to beneficial OTE and in which cases OTE might be unsuitable. Hence, part of this study was to assess the safety and potential benefits of OTE as part of an ERACS-concept.

We hypothesized that implementing an ERACS-protocol with OTE in patients undergoing elective coronary bypass and aortic valve replacement surgery would reduce postoperative length of stay (LOS), improve clinical outcomes, and maintain patient safety compared to the standard approach of delayed extubation. We postoperatively evaluated patient satisfaction for clinical quality control using a structured questionnaire.

## 2. Materials and Methods

In a single-center study at the University Hospital of Regensburg, we compared a prospective RERACS-group (*n* = 114) who underwent elective cardiac surgery between April 2023 and 3 September 2024 adhering to the RERACS concept with a conventionally treated retrospective control group (*n* = 119) from 1 July 2022 to 31 March 2023. The data analysis used electronic patient files, including electronic patient data management systems (Metavision vs. 6, SAP) and other medical records. Inclusion criteria were age 18–75 years, elective coronary bypass surgery or aortic valve replacement surgery, body mass index (BMI) 15–35 kg/m^2^, left ventricular function >30%, normal preoperative respiratory status (O2 saturation >95% or paO2 > 70 mmHg), and pre- and postoperative cardiopulmonary stability. Exclusion criteria were resurgery, emergency surgery, active endocarditis, high risk of postoperative bleeding, and relevant comorbidities (e.g., chronic renal insufficiency requiring dialysis, severe COPD (chronic obstructive pulmonary disease) severe liver cirrhosis, New York Heart Association (NYHA) class IV).

Endpoints were the duration of intensive and intermediate care (LOS ICU, LOS IMC) treatment and length of postoperative hospital stay (LOS hospital). Invasive mechanical ventilation (IMV) time was defined as postoperative mechanical ventilation duration in the intensive care unit (ICU) until extubation. Early extubation was defined as OTE performed in the OR immediately following surgery. Duration of postoperative catecholamine therapy was defined as the time from arrival to the ICU to the end of all inotrope or vasopressor support.

For the detection of postoperative cognitive dysfunction, the nursing staff calculated the Intensive Care Unit Delirium Screening Checklist (ICDSC) [12] once per shift. Likewise, the Numeric Rating Scale (NRS) was evaluated once per nursing shift in the ICU.

For clinical quality assurance, patient satisfaction was evaluated with a structured questionnaire [13]. It contained a total of 12 questions concerning areas of patient satisfaction and the most common patient complaints after general anesthesia (i.e., postoperative nausea and vomiting (PONV), shivering, sore throat, and postoperative cognitive dysfunction). Postoperative evaluation of patient satisfaction was first introduced within the implementation of the RERACS concept and was not yet established in the retrospective control group.

### 2.1. Study Protocol

**Figure 1:**
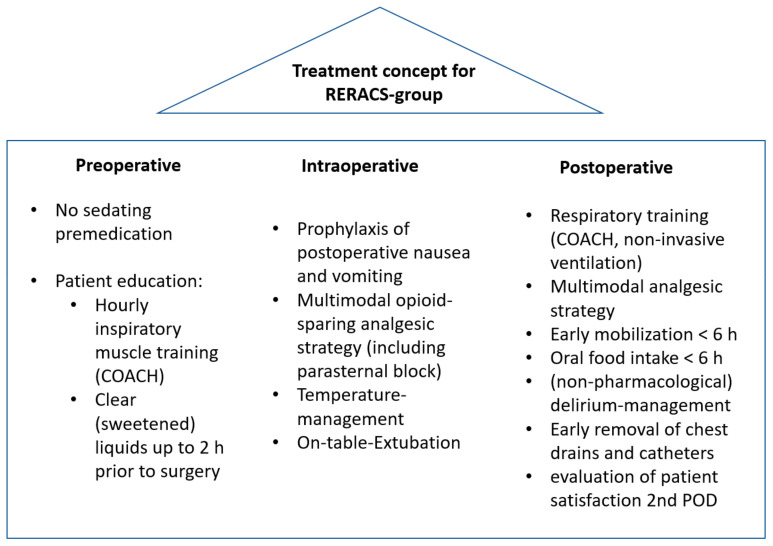
Perioperative treatment concept for RERACS-group.

In accordance with the recently agreed-upon guidelines by the ERAS-Society [5], the French Society of Anesthesia and Intensive Care [6], and the interdisciplinary expert consensus statement [7], we created the RERACS concept. It consists of three perioperative treatment pillars (Figure 1): Before the surgery, the patient’s physiological status was optimized. During the operation, the surgical and anesthesiologic stressors were kept to a minimum, and during the postsurgical phase, implemented measures empowered the patient to commence rehabilitative procedures and re-establish autonomy as early as possible.

#### 2.1.1. Preoperative Optimization

The goal of preoperative patient optimization was to increase muscular fitness and respiratory function before the surgical intervention [14,15]. From the beginning, the patient was integrated into the treatment process to actively contribute to pre- and rehabilitation. As part of the RERACS study patients were introduced to respiratory training exercises presurgery that recommenced postsurgery (hourly training of max. inspiration with a respiratory trainer (COACH)). The instructions for COACH usage and testing of patient compliance were performed at random by the principal investigator, a doctoral candidate, or ward staff. Another principal element of preoperative optimization was minimizing preoperative fasting periods. Patients consumed solids up to 6 h before surgery. Up to 2 h before induction of anesthesia patients consumed clear (sweetened) liquids (one glass (350 mL) of sweetened tea). Sedating premedication was avoided.

#### 2.1.2. Intraoperative Management

During surgery, anesthesiologic and surgical invasiveness were kept to a minimum, and measures were implemented to enhance the patients’ postoperative comfort. Intraoperative anesthesia management was based on previously published ERAS-concepts, the S3-guidelines for analgesia, sedation, and delirium management in intensive care medicine [16], and in-house standards. Necessary preoperative parameters consisted of normal respiratory status with an O2 saturation of >95% or PaO2 > 70 mmHg on arterial blood gas analysis with room air. After induction of general anesthesia with a sufentanil bolus (0,5 µg/kg ABW), propofol (1–2 mg/kg ABW), and rocuronium (0.6 mg/kg ABW), narcosis was maintained with sevoflurane (0.7–0.9 MAC), dexmedetomidine starting at CPB (40–80 µg/h), and remifentanil (0.2–0.75 µg/kg/h). OR staff used standard monitoring, including electrocardiogram, O2 saturation, temperature, invasive blood pressure, and online central venous pressure monitoring. A lung-protective ventilation bundle consisting of low tidal volume ventilation, a PEEP ≥5cm H2O, and a driving pressure <16 cm H2O was applied. The usage of a remifentanil-infusion pump, sufentanil boluses (max. dosage 1 µg/kg ABW for the entire surgery), 1 g metamizole, and parasternal wound infiltration (20 mL ropivacaine 7.5 mg/mL) in accordance with multimodal analgesia enabled pain-free awakening. PONV prophylaxis consisted of dexamethasone (4–8 mg) and ondansetron (4 mg). After weaning of CPB a recruitment maneuver to prevent atelectasis was conducted. The target core temperature was 36 °C or higher. In each instance, the decision of OTE or delayed extubation was created collaboratively by the team based on factors like the course of the surgical procedure and the patient’s hemodynamic situation. If the OTE criteria were met (cardiopulmonary stability, dry situs, no operative concerns), the patient was extubated and then moved to the ICU.

#### 2.1.3. Postoperative Measures

After moving the patient to the ICU, the respiratory training that was introduced presurgery recommenced as soon as possible. If required, noninvasive ventilation (NIV) was introduced. Oral fluid or water ice intake commenced within 6 h after extubation, and a full diet started on the first day after surgery. Another important aspect of the postoperative pillar was the early detection and treatment of postoperative cognitive dysfunction. For detection, both study groups underwent the ICDSC, which was routinely calculated once per nursing shift. Nonpharmacological strategies included early mobilization (within 6 h after surgery), pain management, avoidance of benzodiazepines, patient reorientation, cognitive stimulation, reduction of hearing and/or visual impairment, use of clocks/calendars, and the promotion of a normal sleep-wake circadian pattern. Another parameter that was routinely evaluated once per shift in the ICU is the NRS. A multimodal opioid-sparing analgesic regimen is essential for an early and successful recovery and consists of a continuation of the intraoperatively initiated analgesia following in-house standards. Furthermore, the timely removal of chest tubes and catheters was an important element of the postoperative pillar. The ICU team formally assessed the necessity daily and removed indwelling catheters and chest tubes as early as possible. For clinical quality assurance, patient satisfaction was evaluated with a structured questionnaire [13] on the second postoperative day.

### 2.2. Control Group

The control group received perioperative standard care (Table 1). Induction of anesthesia in the control group was conducted via sufentanil (1 µg/kg), propofol (1–2 mg/kg), and rocuronium (1 mg/kg). We handled the maintenance phase using sevoflurane and intravenous infusion of sufentanil (1 µg/kg/h). Patients in the control group were sedated with propofol (3 mg/kg/h) during transfer and until extubation in the ICU.

### 2.3. Statistical Analysis

Statistical analyses were performed using IBM SPSS Statistics 28 (SPSS Inc.: Chicago, IL, USA). The biometric planning for group size was based on previously published measurements (mean values and standard deviation) and existing hospital data. The power calculation was carried out using the freeware statistical analysis program G*Power 3.1.9.7 (Heinrich Heine Universitty, Düsseldorf, Germany). The effect size d between the comparison groups was calculated by comparing the means and standard deviations in G*Power. The group size n was estimated using the nonparametric test method for 2 independent samples. The alpha error was assumed to be 0.05 and the beta error to be 0.8. The two-sided test was carried out using the Mann–Whitney U test. Based on this, a group size of n = 50 was calculated. Assuming a dropout rate of 20%, this results in a sample size of n = 60 per study group.

The treatment group ultimately included 114 patients and the control group 119 patients. This minor deviation is not expected to substantially impact the statistical power or validity of the study.

Descriptive statistics are reported as mean ± standard deviation (SD) for normally distributed continuous variables or as median with interquartile range (IQR) for skewed variables. In the exploratory data analysis, differences between the treatment groups were assessed using a two-sample *t*-test or, for skewed data, nonparametric Mann–Whitney tests. To analyze frequencies, either the chi-square test or Fisher’s exact test was used. The significance level was set to *p* < 0.05.

## 3. Results

For the control group, 119 patients meeting the inclusion/exclusion criteria during the data collection period of 1 July 2022–31 March 2023 were identified. The RERACS-group contained all patients treated according to the RERACS-protocol from 1 April 2023 to 3 September 2024.

**Figure 2:**
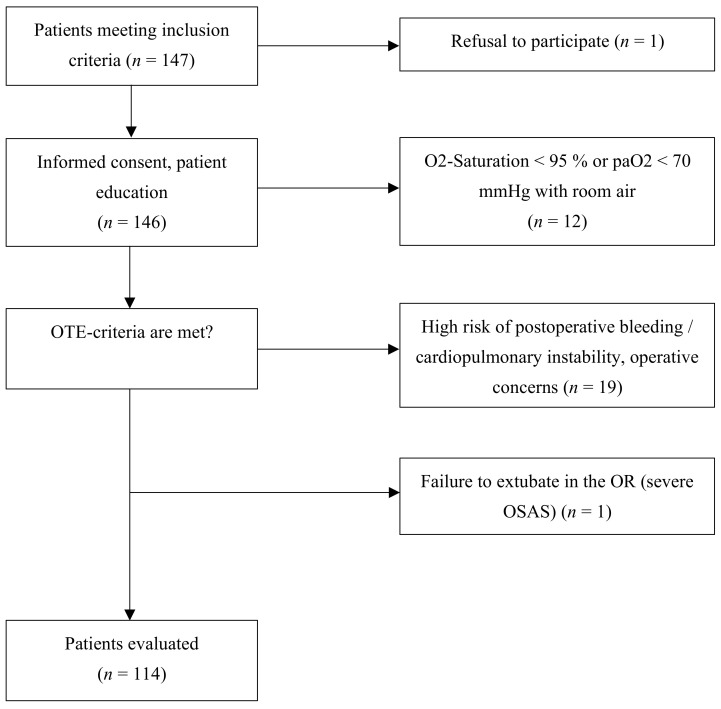
Patient selection flow.

In total, 233 patients were included in the study. Thirty-two ERACS patients had to be excluded (Figure 2). Considering the failure of timely extubation of 20 patients in the RERACS group (one patient due to severe OSAS with a difficult airway and 19 due to high risk of postoperative bleeding or cardiopulmonary instability), we compared 114 RERACS patients with 119 controls.

### 3.1. Primary Results

Table 2 demonstrates a comprehensive list of preoperative and intraoperative characteristics of the participants that could act as potential confounders. Importantly, general variables of the RERACS group did not differ significantly from those of the control group. Furthermore, there were no significant differences between the two groups regarding EuroScore II, age, gender, BMI, total surgery time, and CPB time, which supports a balanced analysis and reduces the risk of bias. The difference in ASA scores was deemed negligible due to the overall comparability of other relevant parameters.

**Figure 3:**
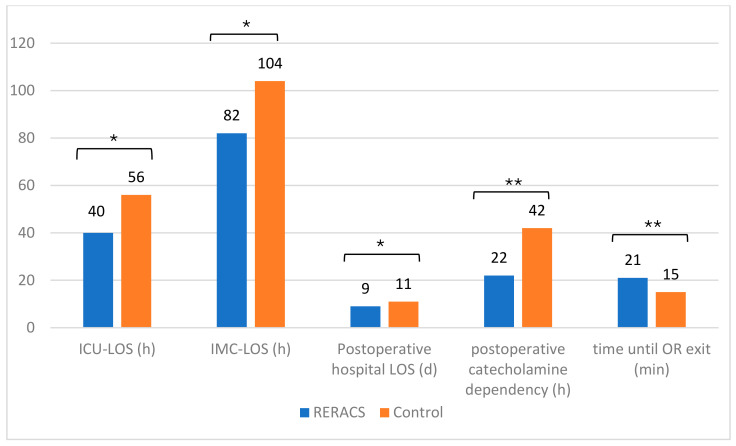
Mean duration of intensive care therapy (LOS ICU), intermediate care therapy (IMC LOS), length of postoperative hospital stay (LOS hospital), catecholamine dependency, and time until OR exit in RERACS and control group. * *p* < 0.05 ** *p* < 0.001.

Table 3 and Figure 3 show ICU-LOS (40 ± 24 h RERACS vs. 56 ± 51 h control group, *p* = 0.005), IMC-LOS (82 ± 69 h RERACS vs. 104 ± 79 h control group, *p* = 0.042), and LOS hospital (9 ± 4 d RERACS vs. 11 ± 6 d control group, *p* = 0.028) were significantly shorter in RERACS patients compared to the control group.

### 3.2. Secondary Results

As Table 4 shows RERACS patients showed significantly faster weaning from catecholamine therapy compared to the control group (22 ± 30 h RERACS vs. 42 ± 48 h control group, *p* < 0.001). Postoperative noninvasive ventilation duration showed no significant difference in RERACS patients compared to the control group (427 ± 1201 min RERACS vs. 738 ± 1989 min control group, *p* = 0.914). No difference was observed in the percentage of patients who required NIV (26.3% RERACS vs. 25.2% control). IMV during postoperative intensive care treatment occurred in one RERACS patient for 1045 min due to a redo-operation for hemostasis (IMV time (min) 720 ± 822 control group, *p* < 0.001). Reintubation due to respiratory complications occurred in two control patients and once in the RERACS group.

As seen in Table 5 the total rate of postoperative complications was lower in the RERACS group (62.3% RERACS vs. 82.4% control group), especially regarding the incidence of postoperative cognitive dysfunction (39.5% RERACS vs. 57.1% control group, evaluated with ICDSC), nosocomial infections (23.7% RERACS vs. 40.3% control group), and decrease in cardiac and respiratory complications, as well as PONV (14.9% RERACS vs. 32.8% control group). The number of patients with an NRS ≥ 4 and the postoperative oxycodone requirements did not differ substantially between the RERACS and the control group.

### 3.3. Other Results and Analysis

For clinical quality assurance, patient satisfaction was evaluated with a structured questionnaire (Table 6). It contained a total of nine questions concerning areas of patient satisfaction and the most common patient complaints after general anesthesia (PONV, shivering, sore throat, postoperative cognitive dysfunction). Patients indicated if statements such as: “I was satisfied with the preoperative patient information”,“I experienced a sore throat or hoarseness after waking up”, and “the administered pain therapy was completely sufficient” was applied completely, somewhat, hardly, or did not apply. Notably, complaints of sore throat/hoarseness (10.5% applies completely), nausea and vomiting (6.3% applies completely), and shivering (1.1% applies completely) were low. Additionally, 90.5% of patients reported pain therapy to be completely sufficient.

## 4. Discussion

The RERACS study aimed to examine the effects of implementing an ERACS concept with OTE versus the traditional approach to delayed extubation in the ICU. Key findings were a reduction in postoperative LOS and complications, especially delirium and PONV. These findings went hand in hand with significantly shorter catecholamine times and high patient satisfaction.

### 4.1. Prehabilitation

The cornerstone elements of prehabilitation were inspiratory muscle training and optimized fasting. Cardiac surgery induces a systemic inflammatory response associated with increased oxygen consumption in the immediate postoperative period. Patients with poor cardiopulmonary reserve are less able to sustain these increased demands, leading to avoidable morbidity and mortality. In patients awaiting cardiac surgery, inspiratory muscle training leads to a decrease in postoperative complications and hospital LOS [14]. This is consistent with our findings, which demonstrate faster pulmonary recuperation seen through shorter NIV time and LOS.

Ljungqvist et al. [17] and Järvelä et al. [18] have shown that carbo-loading (350 mL of a drink high in carbohydrates, e.g., sweetened tea) improved insulin resistance and glucose regulation as well as early onset of gastrointestinal function. This did not cause a higher risk of aspiration [18].

### 4.2. OTE

Early extubation (within 6 h) has become a standard component of ERAS protocols in cardiac surgery, but the rate of OTE differs across centers. In our study, the attempt at OTE only failed in one patient with severe OSAS and a difficult airway.

Longer invasive ventilation times are associated with a higher rate of ventilation-associated pneumonia, dysphagia, and longer hospital LOS, as well as higher morbidity and mortality [19,20], and there is some evidence suggesting that OTE after noncomplex on-pump surgery is a feasible option. Badhwar et al. [8] performed OTE in 165 out of 652 diverse cardiac surgery patients and found that OTE following cardiac surgery was not only safe but also improved outcomes and lowered costs. Our study further substantiated these findings through lowered complication rates, especially in nosocomial infection, cognitive dysfunction, and postoperative nausea and vomiting, as well as significantly shorter ICU-, IMC-, and hospital-LOS.

### 4.3. Delirium

Delirium in the postoperative phase of cardiac surgical care occurs in all ages, affecting between 20% and 80% of patients [20,21]. Since this is associated with higher morbidity and mortality [22], delirium avoidance, detection, and treatment are of utmost importance. For detection, both study groups underwent the ICDSC [11], which is routinely calculated once per nursing shift. Fast-track concepts and a sedative-sparing regimen appear to lower the incidence of delirium [23,24,25]. To further reduce the incidence of delirium, RERACS patients were enabled to regain time and place orientation as early as possible thanks to the adapted anesthesia regime with OTE and the usage of glasses or hearing aids.

### 4.4. PONV

PONV is similarly detrimental to patient satisfaction following surgery as poorly managed pain [26]. Reports of PONV show frequencies varying between 20% and 70%, which is indicative of the multitude of risk factors [27]. In the present study, we could demonstrate that medicative prophylaxis with dexamethasone and opioid-sparing multimodal pain management led to a 20% reduction in the incidence of PONV. This is consistent with previous prospective randomized studies [28] where Champion et al. demonstrated a significant 26% reduction in PONV through the usage of prophylaxis and a significant correlation between pain and PONV.

### 4.5. Pain

Pain results in detrimental physiological responses that may lead to postoperative complications, poor patient experience, and increased risk of chronicity, making it a standard metric for quality of recovery [29]. Opioid-based analgesia has historically held a primary role in the perioperative care of cardiac surgical patients [30]. Although effective in providing analgesia, opioids have numerous adverse effects that impede recovery and create a negative patient experience [31]. Multimodal opioid-sparing analgesia has been shown to reduce opioid requirements, facilitate early extubation, and contribute to a shorter length of stay [32], which is in accordance with the results of our present study. RERACS patients showed high satisfaction with pain therapy and postoperative oxycodone requirements equal to the control group. Especially sore throat was perceived as unpleasant and affected postoperative satisfaction. Given the relatively high incidence of vocal cord injury associated with cardiac surgery, it may be advisable for programs to standardize postoperative screening for oropharyngeal injury and dysphagia, particularly in high-risk patients [19,33].

### 4.6. LOS

As a proxy for morbidity, we chose the duration of intensive care treatment and hospital length of stay. ICU treatment and LOS were both significantly shorter in RERACS patients compared to the control group. To further evaluate intensive care therapy, we examined the duration of postoperative catecholamine dependency. RERACS patients required catecholamine therapy for significantly shorter periods of time, suggesting a more stable cardiovascular situation. Additionally, the study demonstrated that the enhanced recovery process goes hand in hand with high patient satisfaction in postoperative evaluation. As shown in previous studies, the reduced morbidity and LOS also led to “an overall >20% cost reduction […] associated with OR extubation compared with early ICU extubation” despite the cost of additional pre- and rehabilitation [8].

### 4.7. Challenges During Implementation

The main challenges during implementation were caused by the adjustment of staff to the updated workflow and cautiousness during initial implementation and increased drop-out rates. Regular team discussions, clear communication, and the emphasis on interdisciplinary cooperation proved to be essential in ensuring the protocol’s successful implementation. Another factor was organizational circumstances like limited ERACS-trained staff availability. Additionally, a specific patient-related challenge arose when OTE failed in one patient due to severe OSAS, suggesting that the protocol should be adapted on an individual basis.

## 5. Limitations of the Study

The present study faced some limitations. For one, this was an analysis of the first patients that entered our RERACS program compared to a retrospective cohort of standard care with all known limitations of such a study design. Additionally, since this was a single-center study, it does not allow for any generalization. In addition, since the studied patients were not randomized, we cannot rule out that some of the differences are due to selection bias. However, since both studied groups were comparable in age and preoperative risk factors, we are convinced that the differences are primarily driven by the positive effects of the RERACS program with OTE leading to enhanced recovery. The study design allowed for an observation of patients in a relatively short timeframe, reducing the risk of bias. Future studies should include randomized controlled trials and the expansion to a multicenter level as well as the evaluation of patient satisfaction in both the control and study groups to further validate these results. We also recommend future studies on cost and resource implications.

## 6. Conclusions and Implications

The RERACS concept improved patient outcomes, particularly by reducing postoperative cognitive dysfunction, cardiac and pulmonary complications, and nausea/vomiting. Our study indicated that, in elective cardiac surgery, OTE is safe as part of an ERACS protocol and leads to an improved recovery process, including shorter LOS, earlier weaning from catecholamines, and high patient satisfaction. The concept has been shown to not only enhance the patients’ recovery but also has the potential to reduce hospital costs and improve resource allocation due to shorter LOS and lower morbidity.

## Figures and Tables

**Table 1:** Differences between conventional and RERACS concept. ABW: absolute body weight; NIV: noninvasive ventilation; ICDSC: intensive care delirium screening checklist; NRS: numeric rating scale.

	RERACS-Group *n* = 114	Control Group *n* = 119
Preoperative management	Patient education: respiratory training, optimal fasting, no sedating premedication	Usual informed consent
Induction of anesthesia	Sufentanil bolus (0.5 µg/kg ABW), Propofol (1 mg/kg ABW), Rocuronium (0.5 mg/kg ABW)	Sufentanil bolus (1 µg/kg ABW), propofol (1–2 mg/kg ABW), rocuronium (1 mg/kg ABW)
Intraoperative anesthesiologic management	Sevoflurane (0.7–0.9 MAC), remifentanil-infusion pump (0.5 µg/kg ABW/min), dexmedetomidin (40–80 µg/h) Sufentanil boluses (max. dosage 1 µg/kg ABW for the entire surgery), 1 g metamizole, parasternal wound infiltration (20 mL ropivacaine 7.5 mg/mL)	Sevoflurane (0.7–0.9 MAC) Sufentanil-infusion pump (1 µg/kg ABW/h)
PONV-Prophylaxis	Dexamethasone 4–8 mg Ondansetron 4 mg	none
Extubation	On-table extubation	After weaning in the ICU
Postoperative Management	Intensive respiratory training, NIV/high-flow if required Mobilization <6 h Oral nutrition <6 h Delirium screening (ICDSC) and management NRS-Screening and multimodal analgesia Early removal of chest drains and catheters Evaluation of patient satisfaction on the 2nd postoperative day	Delirium screening (ICDSC) and management NRS-Screening

**Table 2:** Patient population.

Parameter	RERACS-Group (*n* = 114)	Control Group (*n* = 119)	*p*-Value
EuroScore II	1.21 ± 0.63; 0.98 (3.36)	1.29 ± 0.98; 0.95 (5.60)	0.558
ASA-Score	3.66 ± 0.52; 4.00 (3)	3.84 ± 0.39; 4.00 (2)	0.006
Male sex	91.2 (104)	85.7 (102)	0.189
Age (years)	64 ± 9; 65 (52)	65 ± 8; 65 (39)	0.146
BMI (kg/m^2^)	27.5 ± 3.7; 27.3 (19.2)	27.4 ± 3.5; 27.3 (15.2)	0.381
Preoperative Ejection Fraction (%)	56 ± 9; 59 (53)	56 ± 8; 58 (38)	0.312
Arterial hypertonus	65.8 (75)	72.3 (86)	0.285
Diabetes mellitus	22.8 (26)	20.2 (24)	0.624
Atrial fibrillation	11.4 (13)	6.7 (8)	0.212
Heart block	6.1 (7)	3.4 (4)	0.317
AVR	28.1 (32)	24.4 (29)	0.521
ACB	71.1 (81)	75.6 (90)	0.429
Operation time (min)	196 ± 40; 192 (227)	210 ± 51; 201 (310)	0.165
CPB time (min)	92 ± 24; 90 (109)	96 ± 29; 94 (162)	0.363
Time until OR exit (min)	21 ± 8; 19 (55)	15 ± 5; 14 (39)	<0.001

Data presented as the mean ± SD and median (range) or in percentage/parameter counts (*n*). AVR: aortic valve replacement; ACB: aortocoronary bypass; MIC: minimally invasive cardiac surgery; ITA: internal thoracic artery; CPB: cardiopulmonary bypass; OR: operating room.

**Table 3:** ICU-, IMC- and total length of stay.

Parameter	RERACS-Group (*n* = 114)	Control Group (*n* = 119)	*p*-Value
ICU-LOS (h)	40 ± 34; 24 (172)	56 ± 51; 40 (346)	0.005
IMC-LOS (h)	82 ± 69; 73 (386)	104 ± 79; 84 (433)	0.042
Postoperative hospital LOS (d)	9 ± 4; 8 (23)	11 ± 6; 9 (50)	0.028

Data presented as the mean ± SD and median (range) or in percentage/parameter counts (*n*). LOS: length of stay.

**Table 4:** Ventilation times and catecholamine dependency.

Parameter	RERACS-Group (*n* = 114)	Control Group (*n* = 119)	*p*-Value
Percentage of patients receiving postoperative IMV	1 (1)	100 (119)	<0.001
IMV time (min)	1 px, 1045 min	720 ± 822; 494 (7252)	<0.001
Percentage of patients requiring NIV	26.3 (30)	25.2 (30)	0.816
NIV time (min)	427 ± 1201; 0 (5783)	738 ± 1989 (14717)	0.914
Postoperative catecholamine dependency (h)	22 ± 30; 12 (159)	42 ± 48; 24 (285)	<0.001
Postoperative oxycodone requirements	23 ± 16; 21 (115)	22 ± 13; 20 (56)	0.524

Data presented as the mean ± SD and median (range) or in percentage/parameter counts (*n*). IMV: invasive mechanical ventilation NIV: noninvasive ventilation. In the RERACS group, one patient received IMV for 1045 min. Redo-operations required the patient to go back to the OR under general anesthesia, e.g., for hemostasis, whereas reinterventions were conducted via a cardiac catheter.

**Table 5:** Postoperative complications.

Parameter	RERACS-Group (*n* = 114)	Control Group (*n* = 119)	*p*-Value
Postoperative complication rate total	62.3 (71)	82.4 (98)	<0.001
Postoperative new atrial fibrillation	21.1 (24)	26.1 (31)	0.410
Nosocomial infection	23.7 (27)	40.3 (48)	0.008
Unplanned reintubation	0.9 (1)	1.7 (2)	0.168
Unplanned redo-operation	2.6% (3)	8.4 (10)	0.059
Unplanned reintervention	0.9 (1)	5.0 (6)	0.030
Nausea and vomiting	14.9 (17)	32.8 (39)	0.002
(subsyndromal) delirium	39.5 (45)	57.1 (68)	0.008
Other	2.6 (3)	9.2 (11)	
Pain NRS > 4	63.2 (72)	62.2 (74)	0.809

Data presented as the mean ± SD and median (range) or in percentage/parameter counts (*n*). NRS: numeric rating scale.

**Table 6:** Patient satisfaction.

Question Topic	Patient Reply			
	Applies Completely	Applies Somewhat	Applies Hardly	Does Not Apply
Satisfied with preoperative patient information	100 (95)	0 (0)	0 (0)	0 (0)
Sore throat/Hoarseness	10.5 (10)	13.7 (13)	14.7 (14)	62.1 (59)
Nausea/Vomiting	6.3 (6)	9.5 (9)	6.3 (6)	77.9 (74)
Shivering	1.1 (1)	3.2 (3)	4.2 (4)	91.6 (87)
Pain therapy was completely sufficient	90.5 (86)	6.3 (6)	1.1 (1)	2.1 (2)
Pain outside surgical site	6.3 (6)	4.2 (4)	7.4 (7)	81.1 (77)
Friendliness of staff	96.8 (92)	1.1 (1)	1.1 (1)	1.1 (1)
Satisfied with quality of patient care	100 (95)	0 (0)	0 (0)	0 (0)
Perceptions during anesthesia	0 (0)	2.1 (2)	0 (0)	97.9 (93)
Delirium/confusion	11.6 (11)	10.5 (10)	10.5 (10)	67.4 (64)

Data presented as percentage/parameter counts.

## Data Availability

The data presented in this study is available on request from the corresponding authors.
